# Therapeutic effect of vascular interventional therapy andaspirin combined with defibrase on cerebral ischemia in rats

**DOI:** 10.3892/etm.2020.8661

**Published:** 2020-04-14

**Authors:** Baoshan Li, Shouke Geng, Yuanli Da

Exp Ther Med 16: 891-895, 2018; DOI: 10.3892/etm.2018.6271

Subsequently to the publication of the above article, the authors have realized that the address for the second-listed author, Shouke Geng, was printed incorrectly (both the name of the work institute and the zip code). Specifically, the work institute should have been featured as ‘Department of Neurosurgery, The Third People's Hospital of Pingdu City’ and the zip code should have been presented as ‘266753’. The corrected author affiliation details for this paper are shown below.

BAOSHAN LI^1^, SHOUKE GENG^2^ and YUANLI DA^3^

^1^Department of Neurosurgery, The Third People's Hospital of Qingdao, Qingdao, Shandong 266041; ^2^Department of Neurosurgery, The Third People's Hospital of Pingdu City, Qingdao, Shandong 266753; ^3^Department of Neuro-Rehabilitation, The Third People's Hospital of Qingdao, Qingdao, Shandong 266041, P.R. China

All the authors agree to this Erratum, and the Editor apologizes to the authors for the inconvenience caused.

